# Supplementary material for: COVID-19 Vaccination Improved Psychological Distress (Anxiety and Depression Scores) in Chronic Kidney Disease Patients: A Prospective Study
Source: Vaccines (Basel). 2022 Feb 16;10(2):299. doi: 10.3390/vaccines10020299 (PMC8878467; doi:10.3390/vaccines10020299)
Supplement: Supplementary file 1 [file vaccines-10-00299-s001.zip › vaccines-1571419-supplementary.pdf]

Supplemetary materials

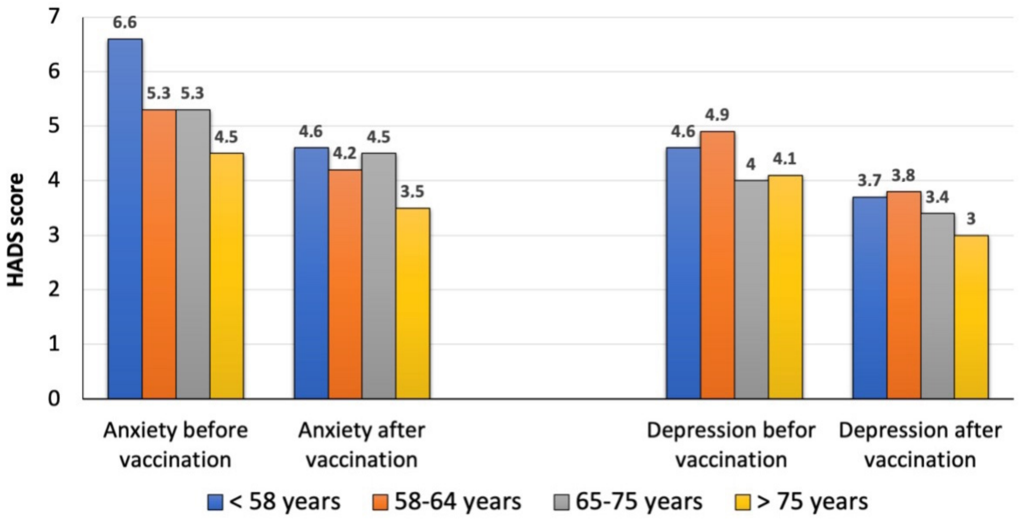

Figure S1 Anxiety score and depression score by groups of age. No significant differences were found between the age groups in both the anxiety and depression scores in the HADS test (p= 0.07 and 0.38 respectively). It is noteworthy that the highest anxiety scores are presented by patients who are in the youngest quartile, while the highest depression scores are found in the range of 56 to 64 years, but that these are within the normal range.

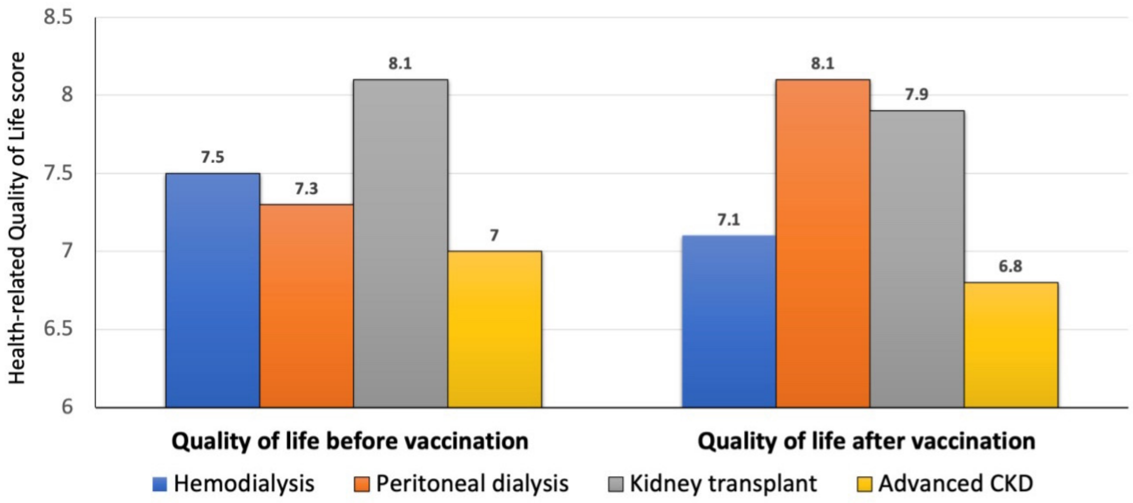

Figure S2 Mean score of self-perceived quality of life before and after vaccination. No significant differences were detected (p=0.12 and p=0.12 before an after vaccination respectively. ANOVA test).

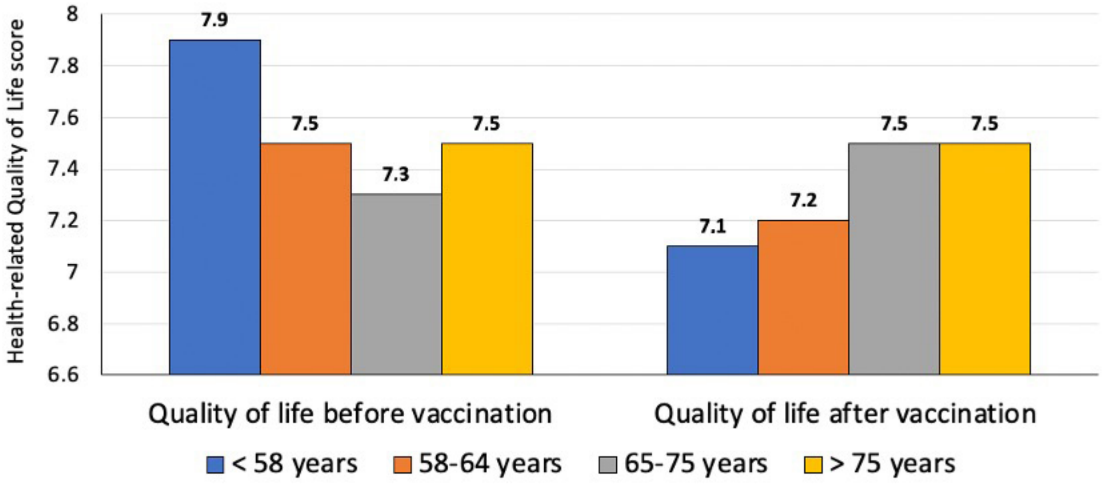

Figure S3. Self-perceived quality of life. No significant differences were detected in the patients' self-perceived quality of life according to age groups, both before vaccination and after vaccination ($p = 0.77$ before vaccination and $p = 0.40$ after vaccination. ANOVA test)
